# Definitive surgical femur fracture fixation in Northern Tanzania: implications of cost, payment method and payment status

**DOI:** 10.11604/pamj.2021.39.126.25878

**Published:** 2021-06-15

**Authors:** Praveen Paul Rajaguru, Honest Massawe, Mubashir Jusabani, Rogers Temu, Neil Perry Sheth

**Affiliations:** 1 The Warren Alpert Medical School of Brown University, Providence, Rhode Island, United States of America,; 2 Department of Orthopaedics, Kilimanjaro Christian Medical Centre, Moshi, Tanzania,; 3 Department of Orthopaedics, University of Pennsylvania, Philadelphia, United States of America

**Keywords:** Health financing, health inequalities, health insurance, access to care, surgery, trauma

## Abstract

**Introduction:**

Kilimanjaro Christian Medical Centre (KCMC) covers major orthopaedic trauma for a catchment population of 12.5 million people in northern Tanzania. Femur fractures, the most common traumatic orthopaedic injury at KCMC (39%), require open reduction and internal fixation (ORIF) for definitive treatment. It is unclear whether payment affects care. This study sought to explore associations of payment method with episodes of care for femur fracture ORIFs at KCMC.

**Methods:**

we performed a retrospective review of orthopaedic records between February 2018 and July 2018. Patients with femur fracture ORIF were eligible; patients without charts were excluded. Ethical clearance was obtained from the KCMC ethics committee. Statistical analysis utilized descriptive statistics, Chi-squared and Fisher’s exact Tests, and Student´s t-tests where appropriate.

**Results:**

of 76 included patients, 17% (n=13) were insured, 83% (n=63) paid out-of-pocket, 11% (n=8) had unpaid balance, and 89% (n=68) fully paid. Average patient charge ($417) was 42% of per capita GDP ($998). Uninsured patients had higher bills ($429 vs $356; p=0.27) and were significantly more likely to pay an advance payment (95.2% vs 7.7%; p<0.001). Inpatient care was equivalent regardless of payment. Unpaid patients were less likely to receive follow-up (76.5% vs. 25%; p=0.006) and waited longer from injury to admission (31.5 vs 13.3 days; p<0.001), from admission to surgery (30.1 vs 11.1 days; p<0.001), and from surgery to discharge (18.4 vs 7.1 days; p<0.001).

**Conclusion:**

equal standard of care is provided to all patients. However, future efforts may decrease disparities in advance payment, timeliness, and follow-up.

## Introduction

The Kilimanjaro Christian Medical Centre (KCMC) is a tertiary referral hospital for the five northern provinces of Tanzania, covering a broad geography and serving a widely disparate population of 12.5 million people. It is the major regional referral center for orthopaedic trauma care with femur fractures being the most common injury encountered (39%) [1]. These injuries typically require open reduction and internal fixation (ORIF) (intramedullary nailing or plate and screw fixation) for definitive treatment. The Lancet Commission on Global Surgery identified affordability as one critical component of surgical care access and based on their complete set of metrics, more than 90% of patients in northern Tanzania lack access to orthopaedic surgical care [2]. Even at KCMC, only 44.5% of patients arriving for care are able to receive definitive orthopaedic surgical intervention when indicated [3].

Previous studies have demonstrated that seeking orthopaedic care in northern Tanzania leads to a significant cost burden on patients and their families [4]. Overrepresentation of insured patients throughout the region´s surgical wards suggests that seeking and receiving surgical care may be linked to payment ability and payment method. It has been shown that the ability to pay alters patient care-seeking behavior [5]. Also, recipients of surgical treatment are more likely to be insured and better able to receive timely care with minimal out-of-pocket costs and lost wages [6].

However, as it pertains to femur fracture fixation, it is unclear what the specific cost burden is and whether payment method (insured vs uninsured) and/or payment status (hospital bill paid in-full or unpaid upon discharge) are associated with care seeking behavior, care delivery and timeliness of care. The aim of this study was to explore associations of payment method (insured or uninsured), and status (paid in-full or unpaid) with episodes of care for femur fracture ORIFs at KCMC.

## Methods

**Study design and setting:** we performed a cross-sectional retrospective review of all orthopaedic procedures performed at KCMC between February 2018 and July 2018, with the aim of identifying all femur fracture ORIF patients. KCMC has a dedicated orthopaedic ward with its own records, which were used to identify potential cases.

**Study population:** all patients having undergone femur fracture ORIF during the retrospective timeframe were eligible; patients without available charts and/or payment data were excluded. There were no exclusions based on demographic characteristics. Patient information was de-identified.

**Data collection:** data was collected from orthopaedic case records which listed procedure names and patient identification numbers. Individual paper patient charts were then requested for confirmation of ORIF, identification of fracture location (i.e. femur or elsewhere), and eventual data collection. De-identified data was collected and entered into a password protected Excel database for statistical analysis.

**Variables:** demographics and injury data including age, sex, mechanism of injury, specific femur fracture location (proximal, middle or distal third), and the open or closed nature of the fracture were collected. Binary data for payment method (insured or uninsured) and payment status (paid in-full or unpaid) were also documented for all patients. Primary outcomes were based on cost, metrics of care delivery and timeliness of delivered care. All data was collected from charts.

**Cost outcomes:** cost outcomes consisted of the bill charged to the patient, amount paid by the patient and whether the patient paid an advance prior to receiving care. Costs were reported in patient charts in Tanzanian shillings (TZS), and were converted to USD (TZS 2,280 per USD as of July 31, 2018).

**Care outcomes:** care outcomes included anesthesia modality (general, spinal or regional blockade), number of radiographs performed, whether post-op imaging was performed prior to discharge, and whether the patient received follow-up care after hospital discharge (binary outcome, yes or no). Intra-operative surgical treatment metrics were also reviewed including anesthesia length (length from induction to procedure end) and procedure length (time from incision to bandage application).

**Timeliness outcomes:** timeliness outcomes included the time from injury to admission, time from admission to surgery, time from surgery to discharge and hospital length of stay (LOS). All timeliness outcomes were reported in days.

**Statistical analysis:** descriptive statistics were utilised to demonstrate the raw outcome values, including demographics, insurance coverage, payment status, and all cost, care, and timeliness metrics. Student´s t-tests were performed to determine differences for all cost and timeliness outcomes based on payment method and payment status, as well as for imaging numbers. Chi-squared and Fisher Exact tests were used to determine differences for imaging before discharge, follow-up care after discharge, and anesthesia modality based on payment method and payment status. Statistical analysis was conducted with Stata IC Release 16 (StataCorp LLC, College Station, Texas). All tests were two-sided with the statistically significant p-value set at 0.05 a priori. As this was an introductory study with a smaller sample size than expected, adjustment was not conducted.

**Ethical considerations:** ethical clearance was obtained from the KCMC Ethics Committee and IRB approval (Research Ethical Clearance No. 2220, Proposal No. 1071) prior to any collection of deidentified retrospective patient data and analysis. All research was in compliance with KCMC research policies and procedures. This study was funded by a travel grant from the University of Pennsylvania Center for Global Health.

## Results

### Patients characteristics

During the study period, a total of 352 ORIF procedures were identified, for which 186 (53%) had available charts. Of this group, 77(41%) were identified as having undergone femur ORIF. Billing data was unavailable for one patient; this patient was excluded from the analysis. Therefore, the remaining 76 (41%) patients met the inclusion criteria. The majority of patients included in this study were male (62, 81.6%), were injured in an RTA (43, 56.6%) or sustained a fall (10, 30.3%) and had a closed fracture (69, 90.8%). Middle third femur fractures were the most common fracture location (29, 38.2%) (Table 1). Most patients did not have health insurance coverage (63, 82.9%), although a majority were able to pay their bill in-full (68, 89.5%). Insured patients were all covered by public schemes, with the majority (12, 92.3%) being covered by the National Health Insurance Fund (NHIF). Most patients (61, 80.3%) paid an advance before receiving surgical care, accounting for between 14.1% to 72.6% of their total bill (95% CI 37.5% to 43.5%). The average bill was $417 (95% CI $369, $465), of which the average patient paid $394 (95% CI $348, $440) (2,280.00 TZS per USD) (Table 2).

**Table 1:** patient demographics characteristics, mechanism of injury, and location of femur fracture

	N or Mean [95% CI]	% or STD
**Sex**		
*Male*	62	81.6%
*Female*	14	18.4%
**Age**	35.5 [31.6, 39.4]	± 17.4 years
**Mechanism of Injury**		
*Road Traffic Accident (RTA)*	43	56.6%
*Fall*	23	30.3%
*Other*	10	13.1%
**Open or Closed**		
*Open*	7	9.2%
*Closed*	69	90.8%
**Fracture Location**		
*Proximal 1/3*	26	34.2%
*Middle 1/3*	29	38.2%
*Distal 1/3*	21	27.6%

**Table 2:** payment method and payment status for entire patient cohort (n=76)

	N or Mean (Range)	% or STD
**Payment Method (Insured vs Uninsured)**		
Covered	13	17.1%
NHIF	12	92.3%
NSSF	1	7.7%
Out-of-pocket	63	82.9%
**Payment Status**		
Paid in-full	68	89.5%
Unpaid Bills Remaining	8	10.5%
**Paid Advance**		
Yes	61	80.3%
No	15	19.7%
**Average Advance Amount (N=61)**	$160 ($110 to $373)	±$70
**Average Advance Amount as % of Total Bill (N=61)**	40.5% (14.1% to 72.6%)	±12.1%
**Charged Bill**	$417 ($76.8 to $1016)	±$215
**Paid Amount**	$394 ($76.8 to $1003)	±$205

### Cost

Patients with an unpaid balance were charged significantly more than patients who paid in-full ($558 vs $400; p=0.049), although statistically they paid the same amount ($339 vs $400; p=0.43). Uninsured patients were significantly more likely to have paid an advance prior to receiving surgical care compared to insured patients (95.2% vs. 7.7%; p<0.001). Unpaid patients had an average remaining balance of $219 (95% CI $95 to $343), or 39.2% of the average bill (Table 3).

**Table 3:** comparison of charges, payments, and advance by payment method and status

	Insured	Uninsured	P-Value	Fully Paid	Unpaid	P-Value
**Bill charged**	$356±$213	$429±$258	0.2650	$400±$207	$558±$246	0.0491*
**Amount paid**	$356±$213	$402±$205	0.4703	$400±$207	$339±$207	0.425
**Paid advance**						
Yes	1 (7.7%)	60 (95.2%)	<0.0001*	53 (87.9%)	0 (0%)	0.138
No	12 (92.3%)	3 (4.8%)		15 (22.1%)	8 (100%)	

### Care delivery

Patients did not differ across most care metrics based on payment method or payment status. However, only 2 (25%) patients with an unpaid balance were seen for post-operative follow-up compared to 52 (76.5%) of patients who paid in-full (p=0.006) (Table 4).

**Table 4:** comparison of care delivery metrics by payment method and status

	Payment Method			Payment Status		
	Insured	Uninsured	P-Value	Fully Paid	Unpaid	P-Value
**Anesthesia**						
GA	0 (0%)	8 (12.7%)	0.337	7 (10.3%)	1 (12.5%)	1.000
SA	13 (100%)	55 (87.3%)		61 (89.7%)	7 (87.5%)	
**X-Rays Performed**	3.6±1.7	4.8±3.2	0.1948	4.6±3.1	5.0±3.1	0.711
**Imaging Prior to Admission?**						
Yes	13 (100%)	57 (90.5%)	0.582	62 (91.1%)	8 (100%)	1.000
No	0 (0%)	6 (9.5%)		6 (8.9%)	0 (0%)	
**Post-Op Imaging Prior to D/C?**						
Yes	13 (100%)	62 (98.4%)	1.000	67 (98.5%)	8 (100%)	1.000
No	0 (0%)	1 (1.6%)		1 (1.5%)	0 (0%)	
**Pt. Seen F/U After D/C?**						
Yes	8 (61.5%)	46 (73.0%)	0.504	52 (76.5%)	2 (25.0%)	0.006*
No	5 (38.5%)	17 (27.0%)		16 (23.5%)	6 (75.0%)	
**Anesthesia Length (minutes)**	156.5±47.6	156.8±50.3	0.9835	158.5±50.2	141.5±43.3	0.3619
**Procedure Length (minutes)**	124.2±38.7	121.5±47.4	0.8467	122.2±46.9	119.9±37.2	0.8920

### Timeliness

No statistically significant timeliness associated differences were observed based on payment method. However, insured patients waited less time across all categories: from injury to admission (11.4 vs. 16.0 days; p=0.30), from admission to surgery (9.5 vs. 13.8 days; p=0.29) and from surgery to discharge (9.1 vs 4.6 days; p=0.075). Insured patients also exhibited a shorter overall LOS (14.1 vs. 22.9 days; p=0.12). Statistical significance was observed between groups with regards to patient payment status. Unpaid patients waited significantly longer across all categories: from injury to admission (31.5 vs 13.3 days; p<0.001), from admission to surgery (30.1 vs 11.1 days; p<0.001), and from surgery to discharge (18.4 vs 7.1 days; p<0.001). Unpaid patients had nearly three times the LOS (48.5 vs 18.2 days; p<0.001) (Table 5).

**Table 5:** comparison of timeliness metrics by payment method and status, days

	Insured	Uninsured	P-Value	Fully Paid	Unpaid	P-Value
**Injury to admission**	11.4±7.9	16.0±14.6	0.3008	13.3±9.6	31.5±28.6	0.0003*
**Admission to surgery**	9.5±7.6	13.8±14.3	0.2911	11.1±8.8	30.1±28.9	0.0001*
**Surgery to discharge**	4.6±2.1	9.1±8.8	0.0750	7.1±6.5	18.4±13.7	0.0001*
**Length of stay**	14.1±8.8	22.9±19.7	0.1196	18.2±12.3	48.5±36.2	<0.0001*

## Discussion

This introductory analysis quantified the cost burden and characterised the payment method and payment status of patients undergoing femur fracture ORIF at KCMC. We found a large cost burden on patients as well as differences in follow-up care and timeliness of care delivery based on payment status. With respect to charges, the direct medical costs observed for femur fracture ORIF were high for the average Tanzanian. For all patients, the average hospital bill was $417; the 2018 GDP per capita in Tanzania was $998. Therefore, direct hospital costs for surgical care of a femur fracture represented nearly half of per capita GDP [7]. This did not include expenses such as travel and foregone income. Previous studies have demonstrated that work-loss associated costs for KCMC outpatients were higher than the cost of care - in excess of $600 per patient. When combined with the average bill, receiving femur fracture ORIF at KCMC may result in catastrophic out-of-pocket expense for the average Tanzanian [4].

While most patients were uninsured, we found that 15.8% of the cohort was insured by NHIF, more than double the coverage seen in the general Tanzanian population (7.1%) [8]. While overall insurance coverage (17.1%) in our cohort was similar to that of the general population (16%), we observed overrepresentation of NHIF coverage. This could be secondary to governmental success in improving patient enrollment [9,10]. Compared to previous findings looking at all surgical conditions at KCMC, overall insurance coverage is actually lower amongst femur ORIF patients (17.1% vs. 41%) [6]. The negative impact on mobility and returning to work may compel patients to seek care regardless of insurance status [11]. This is supported by our patient demographics; the average patient was 35-years-old and over 80% of our cohort was male. This is a key demographic of economic labor, creating an added incentive for seeking definitive treatment [11-14].

Despite high costs associated with care, most patients (89.5%) paid in-full, with uninsured patients paying more overall than insured patients. Patients who were uninsured more often paid an advance prior to receiving ORIF than insured patients (95.2% vs 7.7%), adding to the overall cost. This suggests that an advance functioned as a deposit to demonstrate that the patient could pay [15,16]. This disparity contributes to the larger cost burden on uninsured patients.

Our data demonstrates that regardless of payment method and payment status, patients received the same standard of care. However, patients with unpaid bills were less likely to receive follow-up care after discharge. Following ORIF, continued follow-up and rehabilitation are necessary to ensure adequate healing [17]. A potential reluctance exists on the end of the hospital, unpaid patients, or both for follow-up care. One hypothesis is that patients are worried they will have to pay the remainder of their bill. In addition, a follow-up visit requires additional out-of-pocket expense [18]. After receiving definitive treatment for surgical and/or medical conditions in a variety of LMIC settings, many patients avoid accruing additional costs, missing work, and returning for follow-up [19,20]. Previous studies have found that patients after an orthopaedic injury have experienced disability, loss of employment and lower wages upon discharge [4,21-24]. In addition, other studies have demonstrated an increased risk of treatment abandonment based on payment difficulties [25]. Patients alternatively may be seeking out local, affordable care [26]. An appropriate system is required to ensure patient follow-up.

When analyzing payment status, unpaid patients waited significantly longer than paid patients across all metrics. Previous studies have demonstrated that patients exhibiting difficulty paying were less likely to seek care [27-29]. This contributes to the patient´s decision on whether or not to seek care at KCMC. The availability of local and potentially cheaper options may also cause further delays in presentation to KCMC for patients with an inability to pay [2,26].

For two specific timeliness metrics, admission to surgery and surgery to discharge, unpaid patients also waited significantly longer. It has been described at KCMC that these patients are categorised as D-Still patients - Discharged but still admitted due to an inability to pay [30]. These patients are at risk for absconding due to a fear of debt and being unable to pay in-full [1]. The increased hospital cost burden incentivises the provider side to ensure patient payment at various steps through the care process [25]. Therefore, patients who are unable to pay may be expected to reach a certain level of payment prior to discharge, further prolonging the overall LOS.

Reducing LOS disparities between paid in-full and unpaid patients would decrease the economic burden on patients, increase the ability of KCMC to care for more patients, and improve trainee education by maximizing case-loads [31-34]. This could be done through institutional efforts such as improving insurance coverage through social work programs, establishing extended payment plans and enhancing collaboration with local providers [26,35,36]. These findings naturally feed into the collaborative work currently underway between KCMC and other institutions [37]. Ongoing bilateral work to create an Orthopaedic Center of Excellence is focused on increasing surgical capacity at KCMC and importantly ensuring equal access to care regardless of a patient´s ability to pay.

This study had several limitations. As KCMC did not utilise an electronic medical record during this study, not all were available which may have created a selection bias. Just over half (53%, n=186) of identified ORIF procedures were available. However, this was the most specific and comprehensive data source available. Of our identified ORIF cohort with available paper charts, 41% (n=76) were for patients undergoing femur fracture fixation. This appears to be in line with previous studies demonstrating that femur fractures account for 31-39% of orthopaedic injuries at KCMC [1,3]. While this was not a complete or purely random sampling of retrospective cases, the percentages support a representative sample. The sample size was also limited, but this study was an introductory exploration and will necessitate further study. Regarding generalizability, these findings are specific to KCMC but we hope this analysis may be pursued beyond KCMC.

**Funding:** this work was supported by a small travel grant ($500) from the University of Pennsylvania Center for Global Health.

## Conclusion

Our findings demonstrate that the current system provides equal surgical care, regardless of payment method. However, inadequate payment status results in a large cost burden, a lack of follow-up care and a disparity in timeliness of care delivery. Future research should continue monitoring trends in insurance coverage, quantify the cost burden and explore associations between payment and other surgical procedures. These factors are critical in order to deliver quality care to all patients.

### What is known about this topic

- It is already well understood that musculoskeletal trauma injuries are a common cause of injury and disability, and femur fracture injuries are the most common at KCMC;
- It is also well understood that these injuries require surgical fixation, which may be expensive.

### What this study adds

- This study demonstrates the direct cost burden on patients for surgical femur fracture fixation;
- This study demonstrates differences in the delivery of care to patients based on payment method and payment status, which have not been characterized in any context in sub-Saharan Africa to the best of our knowledge.
